# Oscillatory Motion of Water Droplets Both in Oil and on Superhydrophobic Surface under Corona Discharge

**DOI:** 10.3390/mi13122229

**Published:** 2022-12-15

**Authors:** Qiang Tang, Zongtang Zhang, Jia-Han Zhang, Feiran Tang, Chengjun Wang, Xiaxia Cui

**Affiliations:** 1 School of Artificial Intelligence, Anhui University of Science and Technology, Huainan 232000, China; 2 Collaborative Innovation Center of Advanced Microstructures, School of Electronic Science and Engineering, Nanjing University, Nanjing 210093, China; 3 School of Power and Mechanical Engineering, Wuhan University, Wuhan 430072, China; 4 School of Materials Science and Engineering, Anhui University of Science and Technology, Huainan 232000, China

**Keywords:** corona discharge, oscillatory motion, electrohydrodynamic behavior of water droplet

## Abstract

Charged droplets driven by Coulomb force are an important part of a droplet-based micro reactor. In this study, we realized the rapid oscillatory motion of droplets both in oil and on superhydrophobic surface by injecting charges through corona discharge. Distinct from the oscillatory motion of water droplets under a DC electric field, charge injection can make the motion of water droplets more flexible. A droplet in the oil layer can move up and down regularly under the action of corona discharge, and the discharge voltage can control the movement period and height of the droplet. In addition, the left–right translation of droplets on a superhydrophobic surface can be achieved by injecting charges into the hydrophobic film surface through corona discharge. Two kinds of droplet motion behaviors are systematically analyzed, and the mechanism of droplet motion is explained. The present results could help establish new approaches to designing efficient machines in microfluidics and micromechanical equipment.

## 1. Introduction

In recent years, the manipulation of discrete droplets has increasingly become the focus of research in the field of microfluidics [1,2,3]. Compared to traditional microfluidic technology based on microchannels, the discrete microdroplet system has many advantages, such as less sample consumption, faster mixing speed, less cross-contamination, and easy manipulation [4,5,6,7]. Many discrete microdroplet manipulation technologies based on external fields have been developed, including surface tension [8,9], electricity [10,11], magnetism [12,13], heat [14], light [15], etc. Among these manipulation techniques, electricity is one of the most promising methods, not only because of its shorter response time, but also because of its good compatibility and integration with microfluidic systems [16].

As a new method of droplet manipulation by electricity, contact charge electrophoresis (CCEP) has aroused the interest of academia and engineers in the past decade [17,18]. In contact charge electrophoresis, a particle first charges by contacting the electrode surface in the presence of an electric field, and then the particle can undergo a continuous oscillating motion between the two electrodes under the influence of an electric field [18]. A simple back-and-forth motion through CCEP has been proven suitable for metal particles [19], aqueous droplets [20], and other conductive particles [21]. It is worth noting that CCEP can obtain fast and continuous particle motion with a little energy input [18,19,20,21,22]. This characteristic makes it a cost-effective method for driving microparticles in the field of microfluidics, such as high-speed directional transport of particles in microfluidics channels [21]. However, the particle motion under the control of CCEP lacks flexibility, and the particle motion path is strictly limited to existing between two electrodes [23,24]. In addition, most of the application scenarios are in insulating liquids [18].

In this study, by injecting charge through corona discharge with the needle–plate electrode system to drive droplets, we demonstrate a more flexible CCEP method. Distinct from traditional CCEP, the charge of the droplets in the method described here comes only from contact with the electrode surface. In our experiment, the movement path of the droplet can be controlled by the amount of charge injected into the oil and the superhydrophobic surface by corona discharge. Moreover, the voltage at the tip of the needle determines the movement period of the droplet. The present results will open up new ways of designing efficient machines in microfluidics and micromechanical equipment [25,26,27].

## 2. Experimental Section

**Figure 1:**
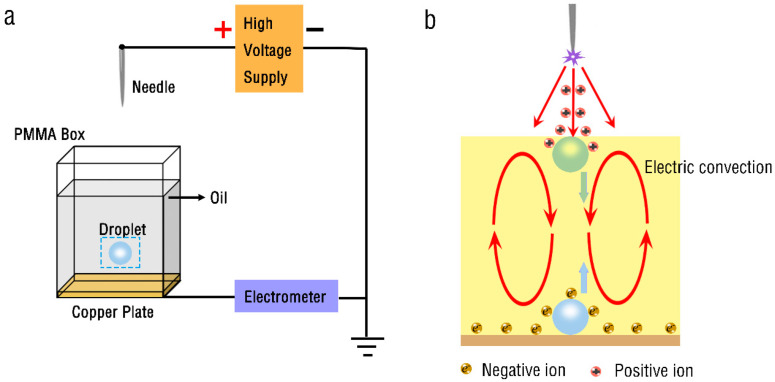
Oscillatory motion of droplets in oil. (**a**) Schematic diagram of the experimental setup. (**b**) Figurative explanation of droplet movement.

The experimental setup of water droplets in oil is schematically shown in Figure 1a. The hollow PMMA (poly methyl methacrylate—PMMA) box is designed to hold oil and water droplets. Its volume is 35 × 10 × 40 mm^3^, and the bottom of the box is sealed with a copper sheet. The oil used in the experiment is olive oil, and the water droplets are deionized water dyed with ink. The tungsten steel needle is placed vertically 10 mm above the center of the hollow PMMA box, which is used as the discharge electrode. The positive pole of high-voltage power supply (Dongwen Corp., DW-P303-5ACCC, Dalian, China) is connected to the needle, and the negative pole is grounded, which can supply voltages varying from 0 kV to 30 kV. The water droplets are deposited in oil with a micro-syringe for each test, and the experiments are conducted at ambient temperature and pressure conditions. Furthermore, to visualize the dynamic behavior of each droplet, a high-speed camera (Phantom, V1612, San Francisco, CA, USA) and a high-definition camera (Sony, A7M3, Tokyo, Japan) were adopted to record the macroscopic behavior of the droplets.

The mechanism of oscillatory motion of droplets in the presence of unipolar ion injection is rather complicated. A figurative explanation of the motion mechanism is shown in Figure 1b. The positive ions are generated because of the locally intense electric field of the needle tip. Positive ions are injected into the oil layer due to the action of the electric field and drive the oil layer to move, forming electric convection [28]. When the water droplet is put into the oil layer, it moves downward and contacts the grounding electrode. At this time, the water droplet is negatively charged and driven upward. On the contrary, when the water droplet moves upward to contact the air/oil interface, it carries positive ions and is driven downward.

**Figure 2:**
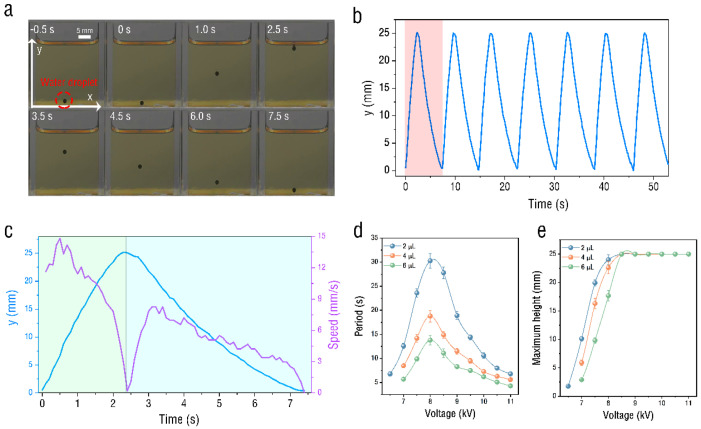
Motion of droplet in oil under corona discharge. (**a**) Images of droplet dynamics in the olive oil with the voltage of 10 kV. (**b**) Time traces of y coordinate under 10 kV. (**c**) The displacement and velocity of the droplet versus the time in one cycle. (**d**) Variation of droplet movement period with voltage. (**e**) Change in maximum height of droplet movement with voltage.

We first examined the case in which the water droplet was in the oil layer. Figure 2 shows the time sequence for the oscillatory motion of droplets with 4 μL volume due to corona discharge. The applied voltage is 10 kV, and the oil-layer depth is 25 mm. As observed in the images in Figure 2a, the droplet is detached from the plate electrode and rises vertically along the y axis. When the droplet reaches the oil/air interface, it moves downward and contacts the plate electrode again. Then, the droplet repeats the above process continuously, as shown in Figure 2b. By extracting the single-cycle data for the displacement versus the time from Figure 2b, the droplet speed can be calculated. The results are presented in Figure 2c. The entire motion process lasts 7.5 s, during which time the droplet motion can be divided into two periods: rising up and descending down. The rising phase of the droplet lasts for 2.4 s, and the maximum speed in the rising phase is 17.82 mm/s, much higher than 8.25 mm/s in the descending down phase. The maximum speeds of both are obtained at the boundary. The equation governing the motion of the water droplet in oil under corona discharge is as follows:

(1) $$m_{e}\frac{dv}{dt}=F_{g}+F_{d}+F_{e}$$

(2) $$F_{g}=\frac{\left(\rho_{w}-\rho_{o}\right)4\pi R^{3}g}{3}$$

(3) $$F_{d}=6\pi \rho_{o}\eta R\nu$$

where $m_{e}=\left(\rho_{w}+0.5\rho_{o}\right)4\pi R^{3}/3$, $m_{e}$ is an effective droplet mass, and $\rho_{w}$ and $\rho_{o}$ are the densities of water and oil. R is the radius of the water droplet. η is the dynamic viscosity of the oil. ν is the velocity of the droplet in a vertical direction. $F_{g}$ is the gravity force. $F_{d}$ is the drag force. $F_{e}$ is the electric field force; it mainly includes Coulomb force and dielectrophoresis force. However, due to the non-uniformity of the electric field and the change of the surface charge of the droplet, the calculation of the electric field force is more complicated. According to the calculation of the acceleration of the droplet, the driving force of the droplet mainly comes from the electric field force.

In addition, the experiment also found that the droplet’s movement period and maximum movement height would change with the discharge voltage, as shown in Figure 2d,e. The data of 2 μL, 4 μL, and 6 μL droplets, respectively, were tested in the experiment. The experiment found that the movement period of the droplet reached its maximum at about 8 kV. At this time, the maximum movement height of the droplet was also closest to the thickness of the oil layer. The larger the volume of the droplet under the same voltage, the smaller the movement period and the lower the movement height.

**Figure 3:**
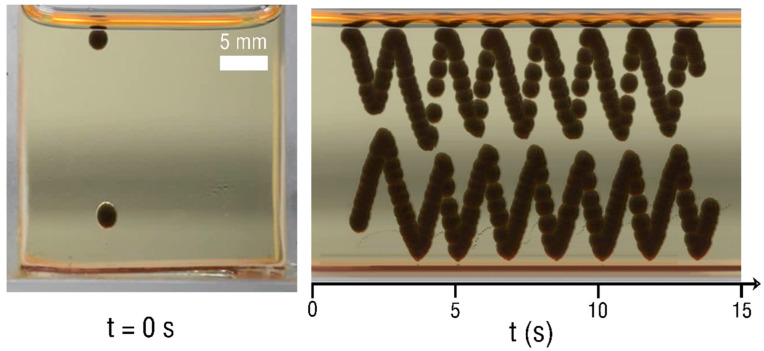
Spatial–temporal images of the synchronized oscillatory motion of twin droplets without coalescence at 10 kV.

In addition to realizing the oscillatory motion of a single droplet, here, we also show the synchronized oscillatory movement of a couple of droplets, as shown in Figure 3. Two droplets with different amplitudes simultaneously exhibit oscillatory synchronous motion in the vertical direction at 10 kV. The two droplets convey electrons by engaging in oscillatory motion between the oil/air interface and the electrode without coalescence. Like a single droplet, the trajectory of the two droplets is confined to the coaxial line. In addition, we also observed that more than two droplets also showed similar synchronous oscillatory movements.

**Figure 4:**
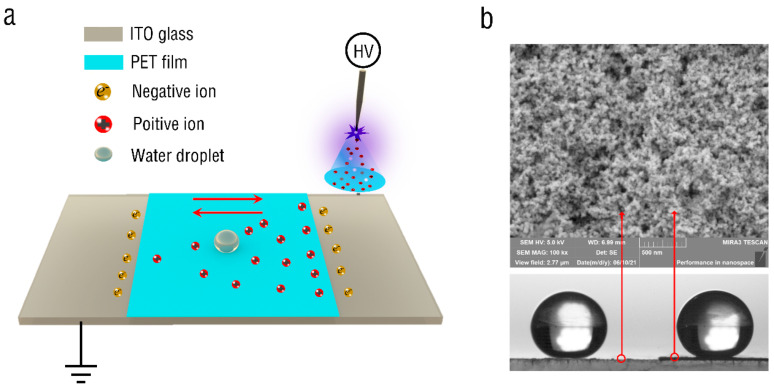
Oscillatory motion of droplets on superhydrophobic surface. (**a**) Schematic diagram of the experimental setup. (**b**) Contact angles and surface morphologies of PET film and ITO glass.

Corona discharge can realize not only the oscillatory motion of water droplets in oil, but also the oscillatory motion of water droplets on the superhydrophobic surface, as shown in Figure 4a. The experiment still adopts needle–plate electrode configuration, in which the plate electrode is ITO glass with a volume of 100 × 100 × 0.5 mm^3^. Furthermore, the center of the plate electrode is covered with PET film, and the size is 100 × 50 × 0.06 mm^3^. The tungsten steel needle is placed vertically 25 mm above the center of the PET film edge. The work plane was treated with commercial nanoparticle coating (Glaco Mirror Coat Zero, Soft99 Co., Tokyo, Japan) to make the PET film and the ITO glass hydrophobic at the same time, as shown in Figure 4b. It can be seen from the figure that the two droplets are on the ITO and the film surfaces, respectively, after hydrophobic treatment, and their surface contact angles are about 158° ± 2°. Through SEM images, it can be seen that there are many nanopores on the hydrophobic surface.

**Figure 5:**
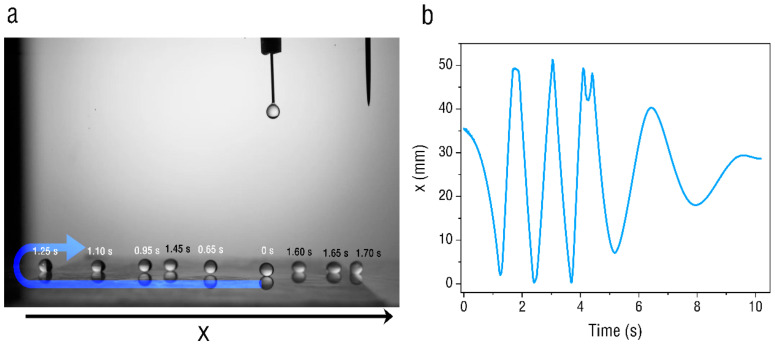
(**a**) High-speed sequential images of the oscillatory motion of a 4 μL water droplet under a voltage of 8 kV. (**b**) Time traces of x coordinate under 8 kV.

Figure 5 presents the droplet dynamics on the hydrophobic surface at the applied voltage of 8 kV. The water droplet drops from the dropper to the PET film surface through the peristaltic pump, and the height of the dropper is level with the tip of the needle. The time when the droplet starts to contact the PET film is recorded as 0 s. Figure 5a shows a sequence of images of a single droplet between the two sides of the PET film. When the droplet starts to contact the PET film, it is positively charged and moves to the left, as it is driven by the electric field on the film surface. Then, the droplet contacts the PET film boundary and starts to rebound, moving to the other side of the PET film. Moreover, the droplet does not move strictly along the x-axis direction due to the uneven distribution of the film’s electric field. Figure 5b shows the droplet position along the x axis as a function of time. It can be seen in the figure that the droplet can contact both sides of the PET film at the beginning and rebound in the opposite direction. With the weakening of the rebound speed of the droplet, the movement of the droplet gradually slows down and finally stops in the center of the PET film.

**Figure 6:**
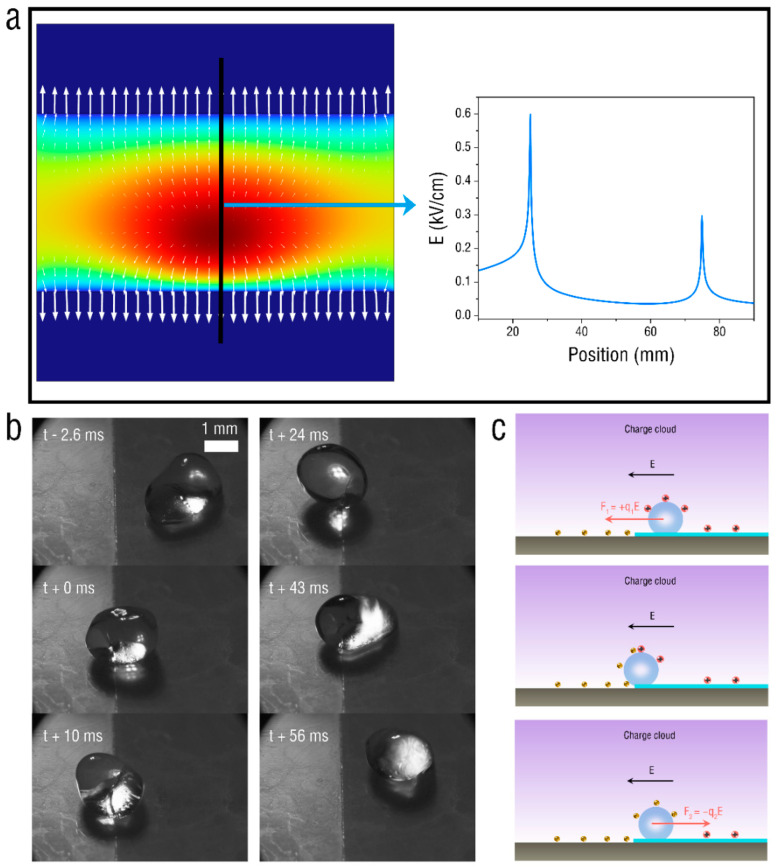
(**a**) Electric field simulation of PET film at voltage of 8 kV. (**b**) Electrohydrodynamic behavior of water droplet at PET-film boundary. (**c**) Mechanism for droplet rebound.

To explore the principle of the oscillatory motion of droplets on the surface of the superhydrophobic PET film, the electric field simulation and high-speed motion capture of droplets are shown in Figure 6. Figure 6a shows the electric field simulation of the PET film at a voltage of 8 kV based on the experimental electrode setup. It can be seen that the electric field distribution on the surface of the PET film is not very uniform. When it comes to the boundary between the film and the ITO glass, the surrounding electric field changes due to the difference in the material dielectric properties. Therefore, there are large electric field barriers on both sides of the film, which can limit the movement of droplets on the film surface. Figure 6b shows the electrohydrodynamic behavior of a water droplet at the film boundary when a corona discharge is applied to the film. When the droplet is close to the film boundary, the droplet stretches towards the film. Then, the droplet contacts the film boundary, and the droplet shrinks and stays at the boundary for a period of time. Finally, the droplet begins to rebound and move away from the film boundary. Here, we can infer the rebound mechanism of droplets at the film boundary, as shown in Figure 6c. The water droplet acquires a positive charge $+q_{1}$ via the charged PET film and rolls towards the grounded ITO glass under the effect of an electrostatic force $F_{1}=+q_{1}E$. Once the droplet contacts the ITO glass, the positive charge on the droplet surface is neutralized and acquires a negative charge $-q_{2}$. It becomes affected by electrostatic force $F_{2}=-q_{2}E$ in the contrary direction [29]. This reversal in the direction of the electrostatic force continuously reverses the direction in which the droplet moves on the film boundary, and eventually leads to the oscillatory motion of the droplet in the air.

## 3. Conclusions

In this study, the fast-oscillating motion of droplets in both the oil and the superhydrophobic surface driven by corona discharge is found in a simple needle–plate electrode configuration. By injecting charge into the oil, the liquid droplets in the oil can realize oscillating movement only by contacting the grounding electrode, and the oscillating period and height can be controlled by the corona discharge voltage. In addition, through the formation of charge distribution and charge barrier on the superhydrophobic surface by corona discharge, the droplets can also realize oscillating movement on the superhydrophobic surface. With the apparent advantages of this novel method, including a simple structure, more flexibility, and a wider application range, we believe that it has great potential to help design more efficient machines in microfluidics and micromechanical equipment.

## Data Availability

Not applicable.
